# Aggregation and Gelation Behavior of Stereocomplexed Four-Arm PLA-PEG Copolymers Containing Neutral or Cationic Linkers

**DOI:** 10.3390/ijms24043327

**Published:** 2023-02-07

**Authors:** Francesca Signori, Jos W. H. Wennink, Simona Bronco, Jan Feijen, Marcel Karperien, Ranieri Bizzarri, Pieter J. Dijkstra

**Affiliations:** 1 Department of Developmental BioEngineering, Faculty of Science and Technology, Tech Med Centre, University of Twente, P.O. Box 217, 7500 AE Enschede, The Netherlands; 2 Consiglio Nazionale delle Ricerche—Istituto per i Processi Chimico-Fisici, CNR-IPCF, Area della Ricerca di Pisa, Via Moruzzi 1, 56124 Pisa, Italy; 3 Department of Polymer Chemistry and Biomaterials, Faculty of Science and Technology, Tech Med Centre, University of Twente, P.O. Box 217, 7500 AE Enschede, The Netherlands; 4 Department of Surgical, Medical and Molecular Pathology, and Critical Care Medicine, University of Pisa, Via Roma 65, 56126 Pisa, Italy; 5 NEST, Scuola Normale Superiore and Istituto Nanoscienze-CNR, Piazza San Silvestro 12, 56127 Pisa, Italy

**Keywords:** poly(lactide)s, stereocomplex, poly(ethylene glycol), PLA-PEG copolymer, hydrogel, aggregation, rheology, solvatochromic fluorophore

## Abstract

Poly(lactide) (PLA) and poly(ethylene glycol) (PEG)-based hydrogels were prepared by mixing phosphate buffer saline (PBS, pH 7.4) solutions of four-arm (PEG-PLA)_2_-R-(PLA-PEG)_2_ enantiomerically pure copolymers having the opposite chirality of the poly(lactide) blocks. Dynamic Light Scattering, rheology measurements, and fluorescence spectroscopy suggested that, depending on the nature of the linker R, the gelation process followed rather different mechanisms. In all cases, mixing of equimolar amounts of the enantiomeric copolymers led to micellar aggregates with a stereocomplexed PLA core and a hydrophilic PEG corona. Yet, when R was an aliphatic heptamethylene unit, temperature-dependent reversible gelation was mainly induced by entanglements of PEG chains at concentrations higher than 5 wt.%. When R was a linker containing cationic amine groups, thermo-irreversible hydrogels were promptly generated at concentrations higher than 20 wt.%. In the latter case, stereocomplexation of the PLA blocks randomly distributed in micellar aggregates is proposed as the major determinant of the gelation process.

## 1. Introduction

It is well known that amphiphilic block copolymers can spontaneously self-assemble into solvents that are selective for one of the blocks [1,2]. The self-assembly may lead to a wide range of structures like micelles, wormlike micelles, vesicles, or polymersomes depending on the relative block length and the solution conditions [3,4,5,6,7,8,9]. At higher concentrations, these nano/mesostructures can transform into “physical” hydrogels, i.e., polymeric networks sustained by extended intermolecular interactions between blocks [10,11]. Owing to their high water-absorbing capacity, hydrogels display a low interfacial free energy in contact with body fluids, resulting in a low adhesion of proteins and cells to adhere to these materials when delivered into the body, mimicking the extracellular matrix and minimizing adverse reactions such as inflammation [12]. For these reasons, hydrogels are a subject of intense investigation and application in several biomedical fields [13,14].

In recent years, particular interest has been given to the self-assembly of amphiphilic block copolymers, composed of biodegradable polyesters as hydrophobic blocks and poly(ethylene glycol) (PEG) as hydrophilic segments [15,16]. In aqueous solutions, self-assembled structures of such copolymers are held together by hydrophobic interactions and are relatively stable. Strategies to increase the stability of polymeric micelles generally rely on chemical crosslinking of either the core or shell [10,17,18]. This approach may successfully facilitate the preparation of stable particles, but it can also lead to a reduced hydrophilicity and/or biodegradability. As an alternative, enhanced stability can also be achieved by dynamic polymer–polymer interactions such as polyelectrolyte interactions [19,20], stereocomplexation [21,22], and hydrogen bonding [23] in either the core or the shell of polymeric micelles. Moreover, it was shown that replacing the hydrolytically labile ester bond between the PLA and PEG blocks with amide groups has a significant effect on gel stability [24].

It Is well known that, upon mixing equimolar solutions of enantiomeric poly(lactide) segments (poly(L-lactide) (PLLA) and poly(D-lactide) (PDLA)), a stable crystalline stereocomplex phase is formed, having a higher melting point than either the homopolymer PLLA or PDLA crystalline phase [22,25,26]. The stereocomplexation between enantiomerically pure poly(lactide) (PLA) blocks has been exploited as a non-covalent crosslinking strategy for the formation of stable PLA-based hydrogels [27,28,29,30,31,32,33]. Examples are the stereocomplexion-driven gelation of PLA-grafted dextrans [34], tri-block [35,36,37,38], penta-block [39], and star-shaped PEG-PLA block copolymers [40,41].

The gelation behavior of PLLA–PEG–PLLA, PDLA–PEG–PDLA and their stereocomplexes has been thoroughly investigated. Aqueous solutions of PLLA-PEG-PLLA may self-assemble into different structures, depending on the molecular weight of the copolymer and the relative block lengths [39,42], from flower-like single micelles to a network of linked micelles, eventually evolving into gel structures according to the so-called “bridging” mechanism [33,37]. In these systems, those relatively weak interactions between the PLA hydrophobic blocks are easily disrupted at higher temperatures, making the hydrogels thermo-reversible. By mixing two enantiomeric polymer solutions (PLLA-PEG-PLLA and PDLA-PEG-PDLA), having comparable polylactide segments lengths, the PLLA and PDLA segments are involved in stereocomplexation, thus acting as stronger linkers between polymer units [33]. However, even these relatively strong stereocomplex interactions can be broken at high temperatures, due to increased molecular motions, depending on the length of the PLA segments, albeit complete sol–gel reversibility is not achievable in most cases, owing to the robustness and insolubility of the PLA stereocomplex crystals formed inside the micelle cores [43].

Contrary to PLA-PEG-PLA amphiphilic block copolymers, research on stereocomplexed PEG-PLA-PEG systems has received minor attention, likely due to the more complex synthetic strategies involved in the preparation of the materials. However, PEG-PLA-PEG systems may be characterized by notable aggregation properties. The temperature-dependent gelation properties of stereocomplexed PEG-PLLA-PEG and PEG-PDLA-PEG amphiphilic block copolymers with molecular block weights of 2000–2000–2000 has been determined [44]. While single-enantiomer solutions did not form gels, mixing enantiomeric solutions provided a thermo-reversible gel, likely due to the reversible coiling (entanglement) and decoiling of PEG segments.

We previously reported on a branched (PEG-PLA-PEG)-like amphiphilic block copolymer, i.e., a four-arm (PEG-PLA)_2_-R-(PLA-PEG)_2_ block copolymer [45]. When R was an aliphatic linear linker, the four-arm (PEG-PLA)_2_-R-(PLA-PEG)_2_ block copolymers afforded thermo-reversible hydrogels, likely due to the intermolecular entanglements of the PEG chains. In contrast, when the R linker in four-arm (PEG-PLA)_2_-R-(PLA-PEG)_2_ block copolymers contained secondary amine groups, which are positively charged at physiological pH, no hydrogels were formed [45]. From these findings, we hypothesized that a different molecular organization occurred in the micelles. The presence of the charged R segments, likely in the micellar corona, effectively hindered PEG entanglements and thereby gel formation [45].

In the present work, the stereocomplexation of enantiomerically pure four-arm (PEG-PLA)_2_-R-(PLA-PEG)_2_ block copolymers having neutral or charged aliphatic R linkers was performed using a toolbox of techniques including Dynamic Light Scattering (DLS), rheology measurements and fluorescence spectroscopy. Stereocomplexation was achieved by mixing in phosphate buffer saline (PBS, pH = 7.2) enantiomerically pure copolymers in a 1:1 (wt:wt) ratio. On the basis of the obtained data, we propose a model for the self-assembly of the micellar aggregates and their possible evolution into stereocomplexed hydrogels taking into account the stereocomplexation of the PLA blocks and by entanglement of the PEG chains.

## 2. Results

### 2.1. Gelation of Enantiomerically Pure Copolymers and Stereocomplexes Thereof

**Figure 1:**
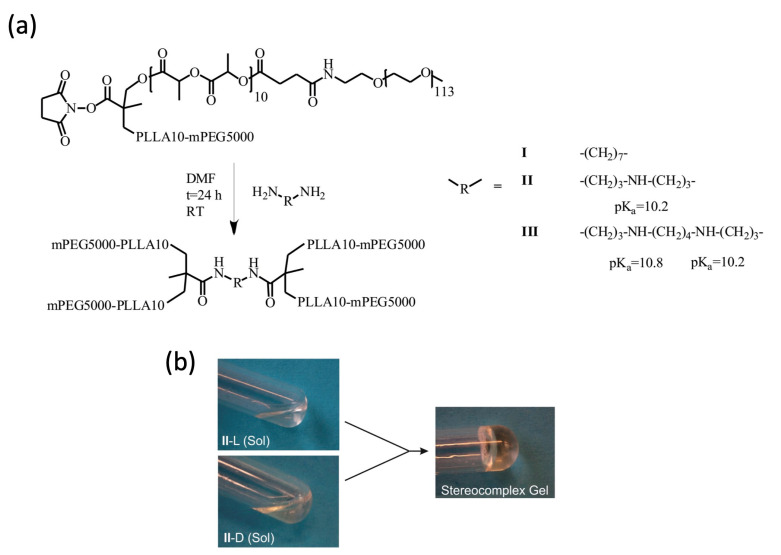
(**a**) Synthetic route for the preparation of four-arm copolymers **I**-L, **II**-L and **III**-L. The synthesis of **I**-D, **II**-D and **III**-D is analog. See Ref. [45] for details. (**b**) Example of gelation in PBS upon mixing 20 wt.% solutions of enantiomerically pure copolymers **II**-L and **II**-D.

Enantiomeric four-arm (PEG-PLA)_2_-R-(PLA-PEG)_2_ block copolymers comprising PLLA blocks or PDLA blocks having a degree of polymerization (DP) of 10 (Mw 1440) and PEG blocks with DP = 113 (Mw 5000) were prepared and characterized as previously described (Figure 1a) [45]. In phosphate buffer saline (PBS, pH = 7.4), copolymer **I**-L, having a central neutral heptamethylene diamide moiety, gave a thermo-reversible gel–sol transition at concentrations above 10 wt.%. Conversely, copolymers **II**-L and **III**-L, containing a charged linking unit with one (**II**-L) or two (**III**-L) secondary amine groups afforded viscous solutions in PBS up to 30 or 40 wt.%, respectively.

In preliminary experiments, we used the vial tilting method at room temperature to assess gelation upon mixing enantiomerically pure amphiphilic block copolymer solutions in PBS to give stereocomplexes. A typical example of transparent stereocomplexed hydrogel is presented in Figure 1b. Hereafter, we shall refer to sc-**I**, sc-**II**, and sc-**III** as the stereocomplexes of copolymers **I**, **II**, and **III**, respectively.

No stereocomplexed hydrogel was formed upon equimolar mixing of **I**-L and **I**-D at concentrations lower than 5 wt.% in PBS. At concentrations between 5 and 10 wt.%, mixing afforded hydrogels. The gelation time (copolymer concentration 5 wt.%) was 36 min, but decreased to 20 min at a copolymer concentration of 10 wt.%. Note that no single enantiomerically pure copolymer solutions could be prepared at concentrations higher than 10 wt.%, because the solutions soon turned into hydrogel, being stable up to 65 °C.

The Critical Gelation Concentration (CGC) through stereocomplexation was found to be around 20 wt.% and 30 wt.% for **II** and **III**, respectively. Interestingly, gelation occurred within 2 min. The faster gelation of sc-**II** and sc-**III** as compared to sc-**I** could have partly resulted from the differences in copolymer concentrations, which are at least two times higher for **II** and **III** compared to **I**. Yet, we may also attribute the faster rate of stereocomplexation of these two copolymers to the presence of positively charged groups in their linking unit R (pK_a_ values of the amine groups are approximately 10.5 [45]), which is believed to favor the exposure of the hydrophobic PLA domains to the water phase and thereby their reorganization in stereocomplexed domains.

### 2.2. Dynamic Light Scattering Measurements

**Figure 2:**
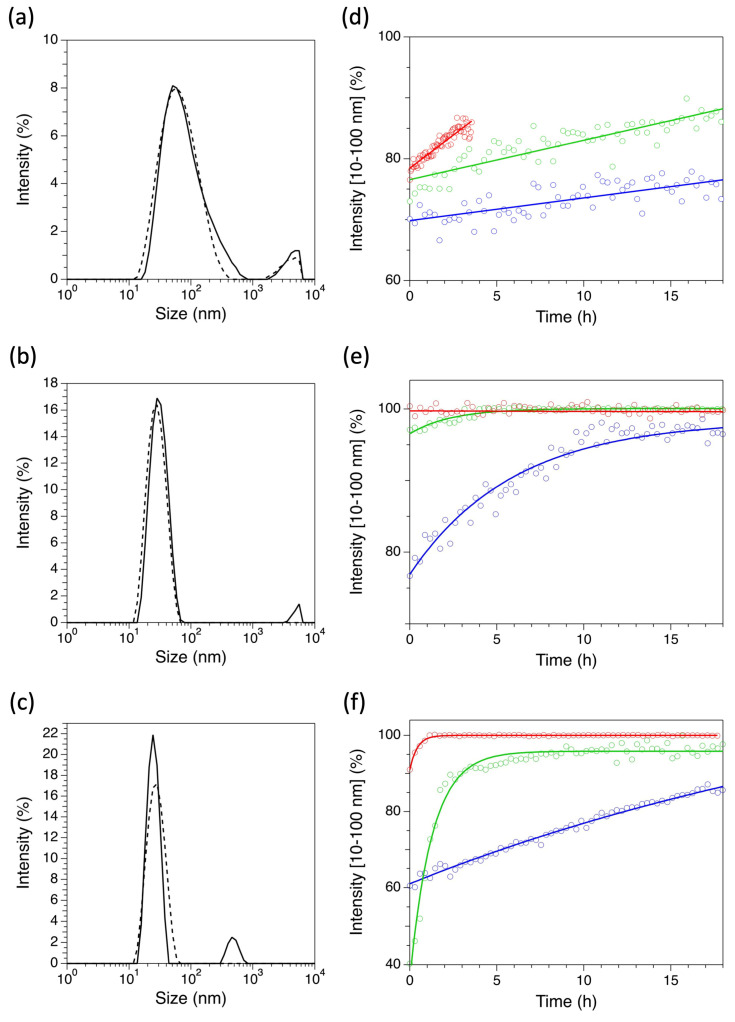
(**a**–**c**) DLS intensity plots of sc-**I** (**a**), sc-**II** (**b**), and sc-**III** (**c**) immediately after mixing (solid line) and after 18 h (dashed line) at 37 °C. (**d**–**f**) Relative DLS intensity (range 10–100 nm) plots after mixing equimolar solutions of copolymers **I**-L + **I**-D (**d**), **II**-L + **II**-D (**e**) and **III**-L + **III**-D (**f**) at 25 °C (blue circles), 37 °C (green circles), and 50 °C (red circles); solid lines represent linear (**d**) or exponential (**e**,**f**) fits to datasets. All experiments were carried out in PBS using 0.3 wt.% of L and D copolymers.

The time-dependent formation of stereocomplexes in PBS was studied under dilute conditions (0.3 wt.%) using Dynamic Light Scattering (DLS). The kinetic effect of three different temperatures, 25 °C, 37 °C and 50 °C, was investigated. Figure 2a–c show the intensity distribution vs. the logarithm of size for sc-**I**, sc-**II**, and sc-**III** (hereafter denoted as “size distributions”) at time zero and after 18 h at 37 °C, the reference temperature for biomedical applications. Figure 2d–f show the kinetic trends of global DLS intensity due to 10–100 nm particles from 0 to 18 h.

Immediately after mixing, solutions of sc-**I**, sc-**II**, and sc-**III** were characterized by the presence of micelles with size in the range 10–500 nm and larger aggregates with size between 500 nm and 10 μm, independently of the temperature (Figure 2a–c, full line). In all conditions, micelles accounted for the largest share of scattered intensity. Considering a typical conversion value of 10^−3^ from intensity % to volume fractions, micelles accounted for more than 99.5% in volume of the aggregate phase for all the stereocomplexes.

Interestingly, after 18 h, the large aggregates of sc-**II** and sc-**III** almost disappeared, leaving only micelles with size < 100 nm (Figure 2b,c, dashed line). Conversely, in this time span, only a fraction of the large aggregates of sc-**I** converted into micelles (Figure 2a, dashed line). In the micellar size range, sc-**I** always exhibited a broader size distribution than sc-**II** and sc-**III**, regardless of the temperature. Additionally, the size at intensity maximum was 50–55 nm for sc-**I** and 28–32 nm for sc-**II** and sc-**III**, with almost no temperature effect.

Raising the temperature inevitably increased the conversion rates of the larger aggregates into micelles (Figure 2d–f). Yet, major kinetic differences could be observed. For sc-**I**, as depicted in Figure 2d, an equilibrium could not be reached in the first 18 h, and the number of micelles linearly increased with time.

For sc-**II**, as depicted in Figure 2e, an equilibrium at 25 °C was slowly reached after approximately 14 h. Upon increasing the temperature to 37 or 50 °C, an almost instantaneous reorganization to the equilibrium distribution of stereocomplexed micelles was observed. This was likely due to faster macromolecular movements allowing a quicker stereocomplexation. The formation of stereocomplexed micelles from **III** showed a peculiar pattern, since three different evolutionary pathways were found, depending on the temperature (Figure 2f). At 25 °C, a linear increase in the intensity plot indicated a slowly occurring exchange in polymer molecules between micelles. At 37 °C, significant rearrangements occurred within the first 2.5 h, and then the equilibrium was reached. At 50 °C, faster molecular motions triggered the complete organization in stereocomplexed structures soon after mixing, returning an equilibrium organization that remained almost constant upon time. Interestingly, at time zero, sc-**II** always showed a larger share of micelles than sc-**III**.

Overall, these findings suggest that the charged linkers (in **II** and **III)** triggered a more homogenous and regular stereocomplexation process, which can evolve in a few hours to an equilibrium characterized mostly by the presence of micelles < 100 nm. This effect was attributed to the larger exposure to the hydrophilic phase of PLA segments in **II** and **III**, which should thermodynamically and kinetically favor the aggregation of enantiomer copolymers into stereocomplexes.

### 2.3. Fluorescence Analysis

To further characterize the stereocomplexation process in diluted solutions, we carried out fluorescence measurements by using the solvatochromic fluorescent sensor Ge1. Ge1 is a green fluorescent protein chromophore analog whose steady-state emission is dependent from local polarity. More precisely, the emission band of the dye, which is in the green region of the optical spectrum, is red-shifted with increasing polarity, and a simple linear relationship holds between the spectral shift and the logarithm of the dielectric constant ε of the dye environment. We previously applied Ge1 to monitor ε in the cellular setting and in Triton-X micelles [46,47].

**Figure 3:**
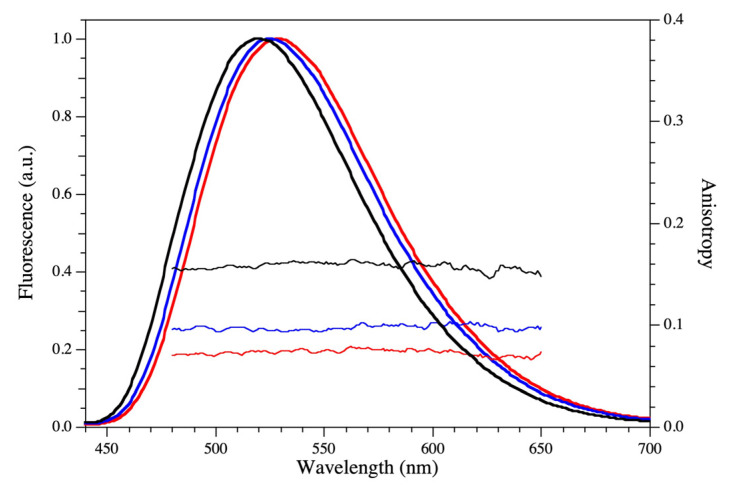
Fluorescence emission (left axis) and anisotropy (right axis) of Ge1 in the 440–700 interval upon 420 nm excitation. Red: **II**-L. Blue: sc-**II** immediately after mixing. Black: sc-**II** 72 h after mixing. Experiments were carried out in PBS at 25 °C using 0.3 wt.% of L and D copolymers.

Ge1 was applied to monitor the formation of sc-**II** at room temperature in PBS (0.3 wt.%). The steady-state fluorescence emission spectrum of copolymer **II**-L was compared to that of sc-**II**, immediately after mixing and after 72 h (Figure 3). In **II**-L, the emission spectrum of Ge1 returned ε = 12, lower than the reported value of 22.4 of oxyethylene palisades in micelles [48]. This finding hints at the localization of Ge1 near the hydrophobic polylactide core of the micelles where it experiences also restricted mobility. This hypothesis was confirmed by measuring the fluorescence anisotropy of the dye in the 480–650 nm range. Indeed, we found that *r* = 0.08; this value is significantly higher than the *r*~0 observed when Ge1 was free to tumble and depolarize in solution.

The emission spectrum of Ge1 was blue-shifted by about 5 nm upon polymer mixing. This reflected a change in dielectric constant from 12 to 9, suggesting an increase in the hydrophobic character of Ge1 environment, likely related to stereocomplexation. This hypothesis was supported by the increase in the dye’s fluorescence anisotropy to about *r* = 0.1, witnessing a more restricted mobility of the dye. After 72 h, **Ge1** signaled an even more apolar environment (ε = 5.7), associated with *r* = 0.16. This finding posited the deep spatial confinement of Ge1 within the core of the equilibrated (vide supra) stereocomplexed micelle. In all cases, the anisotropy values were found to be rather constant across the emission spectral range, suggesting considerable uniformity in the physicochemical features experienced by Ge1 in the monomer, as well as in the stereocomplexed solutions.

### 2.4. Rheology Measurements

**Figure 4:**
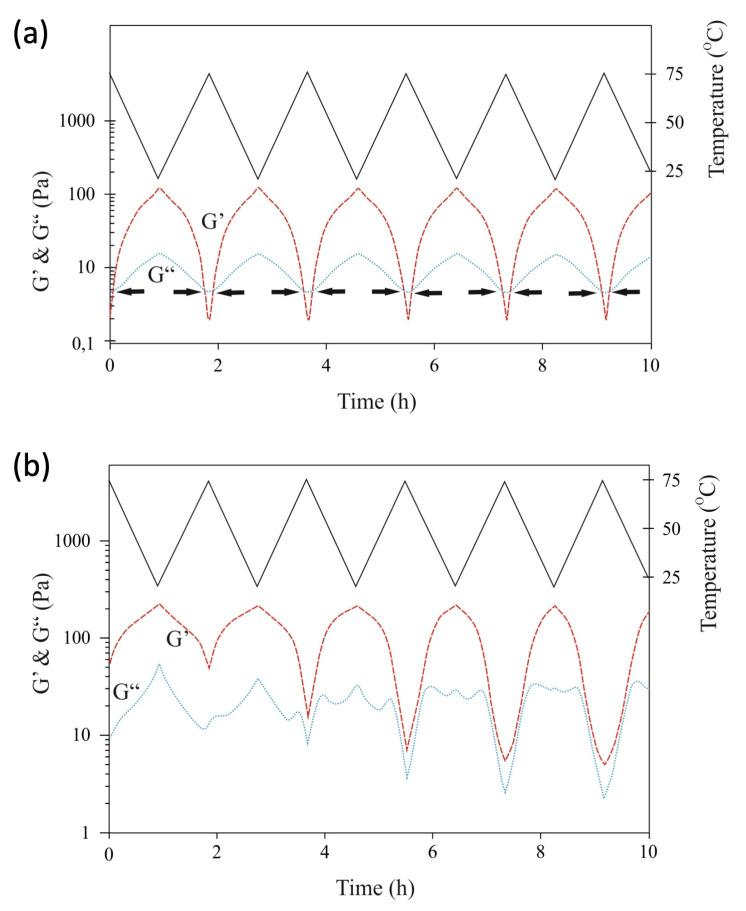
Temperature-dependent storage (G′) and loss (G″) moduli of sc-**I** (**a**) and sc-**II** (**b**) hydrogels upon heating to 75 °C and subsequent cooling to 25 °C (solid grey line). Arrows in (**a**) indicate the sol-gel and gel-sol transitions. In both cases the solutions were prepared in PBS, pH = 7.4. Concentrations: sc-**I,** 10 wt.%; sc-**II**, 20 wt.%.

To further characterize the properties of sc-**I**, sc-**II**, and sc-**III** at high concentrations in PBS, the storage (G′) and loss (G″) moduli of the gels were determined by oscillatory rheology experiments at temperatures between 25 and 75 °C (Figure 4).

The hydrogel from sc-**I** (10 wt.%) showed a gel–sol phase transition on heating, with a critical temperature of gel-to-sol transition (CGT) of 65 °C (Figure 4a). Very remarkably, the CGT of sc-**I** is analogous to the value found for its precursor **I** [45]. Additionally, for both **I** and sc-**I**, the gel–sol transition was fully reversed upon cooling, indicating that the stereocomplex and the enantiomer gels are both thermo-reversible systems. Yet, sc-**I** displayed a similar loss modulus but a higher storage modulus of 123 Pa compared to the storage modulus of **I** (32 Pa, [45]), indicating the formation of a stronger hydrogel upon stereocomplexation.

These findings suggest that the reversible sol–gel transition was predominantly due to PEG entanglements, with a minor contribution of stereocomplexation. This hypothesis explains the observed absence of gel formation at the same concentration (10 wt.%) for **II**-L, **III**-L. Indeed, **II**-L, **III**-L contain a central cationic moiety that is believed to favor the exposure of the PLA segments to the hydrophilic phase, whereas PEG entanglements are likely stronger in neutral **I**-L, for which the phase separation between the hydrophobic and hydrophilic blocks is enhanced.

As already shown by the vial tilting method, sc-**II** and sc-**III** hydrogels were promptly (<2 min) formed upon mixing enantiomeric polymer solutions at concentrations above 20–25 wt.%. Rheology experiments showed that a network remains present in the temperature range of 25–75 °C because G′ was always higher than G″. Two regimes, depending on the temperature, could be identified. Below approximately 50 °C, the G′ values ranged between 100 and 200 Pa and—after a a few transient cycles—G″ stabilized to a value of 20–30 Pa. Larger differences were observed above 50 °C. After the transient, G′ and G″ values decreased to approximately constant values of tanδ (G″/G′). Overall, this behavior could be attributed to a sol phase unable to reorganize into a micellar or aggregate solution and retaining some 3D crosslinks, likely stabilized by hydrophobic interactions and PEG entanglements.

## 3. Discussion

The gelation behavior of ABA triblock copolymers, where A is a hydrophilic block and B a hydrophobic, aggregation-prone block, is thought to occur via the so-called non-bridging mechanism [37,49]. ABA yield star-like micelles in aqueous media at low concentrations. When the concentration is increased, gelation may occur mostly as result of interactions between the A segments. This model fully rationalizes the micellar and rheological behavior of enantiomeric pure triblock copolymer **I** [45]. However, the presence of a central linker whose hydrophilic/hydrophobic balance can be modified by the incorporation of protonatable amine group, as well as the formation of a stereocomplexed PLA phase by mixing enantiomeric copolymers, afforded an interesting way to modulate the properties of the resulting hydrogel.

The observed kinetics of stereocomplexation in micelles were particularly informative. For all samples, we found that micelles consisting of aggregates of 10–500 nm and larger were formed upon mixing of enantiomeric copolymers in PBS at pH 7.4. Changes in the polarity of the environment experienced by the environmentally sensitive probe Ge1, as well as the restriction of its molecular mobility, witnessed the prompt formation of a more hydrophobic PLA phase as a result of stereocomplexation for the representative sample sc-**II**. The particle dispersions evolved with time, with progressive conversion of the larger aggregates into micelles <150 nm, and the temperature inevitably increased the rate of this process. These findings are in agreement with a general mechanism of aggregation into micellar stereocomplexes, in which de-entanglements of PEG chains and de-aggregation of hydrophobic domains of enantiomerically pure copolymer solutions thermodynamically balance the formation of new micellar aggregates having a partly stereocomplexed core. Higher solution temperatures enhance molecular motions, thus favoring de-aggregation and de-entanglement processes, and therefore raising the rate of stereocomplex formation. However, significant differences were observed between sc-**I** and sc-**II**/sc-**III**. sc-**I** was found to yield heterogenous aggregates, which very slowly evolved to a rather broad micellar dispersion peaked around 50 nm. sc-**II** and sc-**III** gave less heterogenous nanodispersions at time zero, and evolved much faster to an equilibrated, narrower micellar distribution with a peak at around 30 nm.

**Figure 5:**
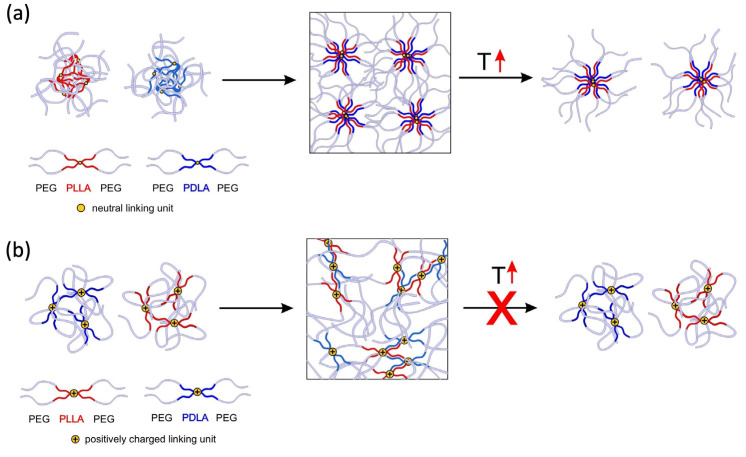
Model of hydrogel formation for block copolymers **I**-L **and I**-D (**a**) and block copolymers **II**-L and **II**-D and **III**-L and **III**-D (**b**).

The stark difference between sc-**I** and sc-**II**/sc-**III** observed in DLS measurements at low polymer concentration (0.3 wt.%) emerged even more significantly in the rheological measurements of stereocomplexed gels at high concentrations (10–20 wt.% in PBS) and temperatures between 25 and 75 °C. The thermo-reversibility of the sol–gel transition of sc-**I** as compared to the irreversible pattern displayed by sc-**II** and sc-**III** were interpreted in light of the different nature of the PLA domains. Indeed, due to the presence of charged segments, the hydrophobic domains in **II** and **III** are expected to be relatively small and more exposed to the aqueous medium in the enantiomerically pure copolymer micelles, therefore being more readily available for stereocomplexation. This hypothesis was cast into a model of the gelation mechanism of the stereocomplexes (Figure 5), which built over our previous findings on enantiomeric copolymers alone [45]. Upon mixing **I**-L and **I**-D, micelles with partly stereocomplexed hydrophobic domains were formed. According to the non-bridging mechanism, thermo-reversible gelation occurs due to the formation and breaking of entangled PEG chains (Figure 5a). Hydrogel formation in the case of copolymers **II** and **III** occurred through a different mechanism (Figure 5b). At room temperature, aqueous solutions of the single enantiomers of copolymers **II** and **III** are in a sol state up until high concentrations. Upon mixing, e.g., **II**-L and **II**-D at a concentration of at least 20 wt.%, a hydrogel is formed by the occurrence of small stereocomplexed domains that act as crosslinks, and which are thermo-irreversible. Gelation in this case is kinetically determined.

## 4. Materials and Methods

### 4.1. Materials

L-lactide (L-LA) and D-lactide were purchased from Purac (Gorinchem, The Netherlands). 2,2-Bis(hydroxymethyl)propionic acid (bis-MPA) was obtained from Acros (Geel, Belgium). Tin (II) 2-ethylhexanoate (Sn(Oct)_2_), succinic anhydride, N-hydroxysuccinimide (NHS), N,N′-dicyclohexylcarbodiimide (DCC), mesyl chloride, deuterated chloroform (CDCl_3_), aqueous ammonia (25%), glacial acetic acid, 1,7-diaminoheptane, norspermidine and spermine were obtained from Sigma-Aldrich (Zwijndrecht, The Netherlands). Methoxy-hydroxy poly(ethylene glycol) with a molecular weight of 5000 g/mol (mPEG5000-OH) was purchased from Fluka (Buchs, Switzerland). Triethylamine and 4-dimethylaminopyridine (DMAP) were acquired from Merck (Darmstadt, Germany). All other solvents were obtained from Biosolve (Valkenswaard, The Netherlands). Dichloromethane and toluene were dried over calcium hydride (Aldrich) and sodium wire, respectively, and distilled prior to use. All other chemicals were used as received.

### 4.2. Methods

*Polymer synthesis:* the four-arm PLLA-PEG block copolymers and four-arm PDLA-PEG block copolymers were prepared as described previously [45].

*Vial tilting*: Block copolymers were dissolved in PBS. Solutions (250 µL) containing equimolar amounts of the single enantiomers were mixed and gently shaken until a gel was formed. The time to form a gel (denoted as gelation time) was determined using the vial tilting method. No flow within 1 min upon inverting the vial was regarded as the gel state. For each sample the experiments were performed in triplicate.

*Rheology*: Rheology experiments were performed with an Anton Paar Physica MCR 301 rheometer with flat-plate geometry (20 mm diameter, 0.3 mm gap) in oscillating mode. Enantiomeric pure copolymers were dissolved in phosphate buffer saline (PBS, pH 7.4) at 10 wt.% (**I**) or 20 wt.% (sc-**II** and sc-**III**) concentrations, mixed with 1:1 stoichiometric ratio. After gelation, the materials were kept at 4 °C overnight prior to measurement. To prevent evaporation of water a solvent trap was interfaced to the rheometer. A pre-shear was applied for 10 s, after which the gel was made it possible to equilibrate for 10 min. Subsequently, the hydrogel was heated to 75 °C at 1 °C.min^−1^, and then cooled to 20 °C at 1 °C.min^−1^. The storage modulus G′ and the loss modulus G″ were monitored applying a frequency ω of 1 Hz and a strain γ of 1% to ensure that measurements were performed in the linear viscoelastic range of the hydrogels. After several heating and cooling cycles, a frequency and amplitude sweep were performed at ω 0.01–10 Hz (γ = 1%) and γ 0.01–10% (ω = 1 Hz) at 20 °C, to confirm that the applied frequency of 1 Hz and a strain of 1% were within the linear viscoelastic range.

*DLS*: Dynamic light scattering experiments were performed on 0.3 wt.% solutions of stereocomplexes using a Malvern zetasizer 4000 (Malvern Corp., Malvern, UK), and a laser wavelength of 633 nm, adopting a 90° detection geometry. The CONTIN method was applied for data processing. All solutions made it possible to equilibrate at each measuring temperature for 15 min. Kinetic experiments on stereocomplexation were performed by determining the relative intensity of the peak for sc-**I** at 50 nm and for sc-**II** and sc-**III** at 32 nm by mixing 0.3 wt.% solutions of enantiomeric copolymers (equimolar amounts) in PBS. The changes in the scattering intensities were determined every 15 min for 15 h at 25, 37 and 50 °C.

*Fluorescence measurements:* Fluorescence intensity and anisotropy measurements were carried out with a Cary Eclipse fluorometer (Varian, Palo Alto, CA), using 2 nm bandpass, 1 nm step size and 0.2 s integration time. Ge1 was dissolved in the sc-**II** solution in PBS (0.3 wt.%) at 1–5 μM from a concentrated THF solution (10–50 mM). Excitation was carried out at 420 nm.

The solvatochromic behavior of Ge1 emission is associated with a linear relationship between the logarithm of the local dielectric constant ε and the “generalized polarization” GP calculated from the fluorescence intensities collected in the “hydrophobic” range 480–525 nm (F_L_) and the “hydrophilic” range 540–580 nm (F_H_) according to:

(1) $$\mathrm{GP}=\frac{F_{H}-F_{L}}{F_{H}+F_{L}}$$

Since GP is a concentration-independent (“ratiometric”) optical response, its dependence on the dielectric constant is universal, and can be retrieved by a calibration with media of known dielectric constant. GP vs. ε calibration was carried out according to the procedure reported in [46]. The dielectric constant of the local environment experienced by Ge1 in the polymer solution was obtained from the GP measurement by means of the GP vs. ε calibration.

## 5. Conclusions

Four-arm stereocomplexed (PEG-PLA)_2_-R-(PLA-PEG)_2_ hydrogels were prepared by mixing PBS solutions of enantiomerically pure copolymers with opposite chirality. Gel formation was driven by stereocomplexation of PLA blocks, since single enantiomer solutions did not form a gel at similar concentrations. When R was a heptane linker (sample **I**), a thermo-reversible gel was obtained having relatively slow gelation rate and occurring at around 10 wt.%. For this material, gelation at lower temperatures was attributed to the formation of PEG entanglements. Incorporating cationic moieties in the central linker (R, sample **II** and **III**) triggered a pronounced effect on the gelation behavior. Indeed, enantiomeric mixtures of these copolymers afforded stereocomplexed gels at relatively high concentrations (20–25 wt.%), which behaved as thermo-irreversible. The gelation mechanism of these copolymers was attributed to the formation of intermolecular stereocomplexed domains, randomly dispersed in the gel, acting as crosslinks.

## Data Availability

Data contained within the article is available on request from the authors.
